# Exposure to Potentially Harmful E-Cigarette Emissions via Vape Tricks: Protocol for a Mixed-Methods Study

**DOI:** 10.2196/12304

**Published:** 2019-04-15

**Authors:** Robert Furberg, Alexa M Ortiz, Michelle McCombs, Margaret Cress, Jonathan Thornburg, Jessica K Pepper, Youn Ok Lee

**Affiliations:** 1 Digital Health and Clinical Informatics RTI International Research Triangle Park, NC United States; 2 Air Quality and Exposure RTI International Research Triangle Park, NC United States; 3 Center for Health Policy Science and Tobacco Research RTI International Research Triangle Park, NC United States

**Keywords:** electronic nicotine delivery systems, vaping, human exposure modeling, digital health, wearable electronic devices

## Abstract

**Background:**

The number of adolescents and adults using e-cigarettes, referred to as *vaping*, has dramatically increased. E-cigarettes can be used to perform *vape tricks* by inhaling and exhaling the e-cigarette aerosol in patterns to create visual effects or large clouds. To create these effects, the puffing patterns associated with vape tricks may be different than standard ad-lib e-cigarette usage. The prevalence of vape tricks and the harm associated with exposure to e-cigarette emissions when performing vape tricks is currently unknown.

**Objective:**

Our objectives are to characterize duration, heart rate, respiratory rate, tidal volume, minute volume, and physical activity metrics associated with the performance of vape tricks and to characterize the emission of e-cigarettes when performing vape tricks in a manner suitable to inform novel exposure modeling.

**Methods:**

The study will recruit e-cigarette users with a history of performing vape tricks. Data collection will occur in two different sessions. In the first session, participants will be asked to puff on their e-cigarette as they normally would for 20 minutes. The second session will be a vape tricks session, where users will be asked to perform a series of up to five different vape tricks with their e-cigarette. Data will be collected through screener surveys, in-person interviews, video recordings, a personal exposure monitor, and a biometric garment.

**Results:**

Data analysis is pending and scheduled to take place in the fall of 2019.

**Conclusions:**

This study will be used to assess the feasibility of using a biometric garment to complement environmental and observational data. The approach may provide greater insight into the health risks of performing vape tricks compared to typical e-cigarette use.

**International Registered Report Identifier (IRRID):**

DERR1-10.2196/12304

## Introduction

Adolescent and adult use of e-cigarettes, referred to as *vaping*, has increased dramatically since e-cigarettes were introduced to the US market in 2007 [1,2]. In 2016, 15% of US adults reported having tried e-cigarettes and 3% used them some days or every day at the time of the study [3]. Among US youth that same year, 6% of 8th graders, 11% of 10th graders, and 13% of 12th graders reported vaping in the past 30 days [4].

E-cigarette use poses a variety of possible health harms. Most, though not all, e-cigarettes contain nicotine, a substance that impairs adolescent and fetal brain development and can be toxic through ingestion of, or dermal exposure to, high concentrations [5]. Adolescent e-cigarette use may lead to future initiation of cigarette smoking [6]. Although e-cigarettes generally produce fewer harmful constituents than combustible tobacco, their aerosol can contain formaldehyde, acrolein, diacetyl, and other harmful chemicals [7,8]; advanced vaping devices, which heat the liquid inside the device to a higher temperature, produce greater quantities of these chemicals [9]. In some in vitro and in vivo studies, exposure to e-cigarette liquids and aerosols has been linked to reduced cell viability, proinflammatory responses, and impaired immune response [10,11]. Long-term and short-term health effects in users are unclear [12-14]. One potential benefit is that e-cigarette use could help smokers quit, but the evidence about whether e-cigarettes can serve as successful smoking cessation products is extremely inconsistent [15,16].

Youth and adults report using e-cigarettes for a variety of reasons, including curiosity, appealing flavors, friends’ use, and the desire to use a product that is less harmful than cigarettes [17,18]. Youth and young adults also report using e-cigarettes to perform *vape tricks*, where they inhale and exhale e-cigarette aerosol in patterns to create visual effects or attempt to create the largest cloud of aerosol [19,20]. In a recent, nonrepresentative survey of adolescents recruited through social media, 78% reported having tried performing vape tricks and the majority had watched them being performed in person (84%) or online (74%) [21]. Most tricks are performed using advanced devices that reach high temperatures, producing greater levels of harmful chemicals [9]. Performing vape tricks may also involve different puffing patterns than ad-lib use. If vape tricks require deeper or longer inhalations, this pattern could potentially result in increased exposure to harmful emissions.

The population-wide prevalence of vape trick behavior is not yet known, nor is the exact level of harm associated with vape tricks. In an attempt to begin addressing this gap, this paper describes the protocol of a feasibility study where the primary aim is to collect and compare the heart rate, breathing rate, and activity metrics associated with performing vape tricks alongside the metrics of standard ad-lib e-cigarette usage. A secondary outcome consists of using initial study results to program a smoking machine that will be used during subsequent study phases.

## Methods

### Approval

This study protocol underwent review and received approval by RTI International’s Office of Research Protection Institutional Review Board. Additional modifications or changes to the proposed protocol will be submitted as a separate amendment. Breeches or adverse events will be documented by the research team and reported to the Institutional Review Board.

### Participant Eligibility Criteria

The target population for this study is current e-cigarette users between 18 and 29 years of age with a product usage history of e-cigarettes to perform vape tricks at least once during the past 3 months, experience performing at least two different tricks, performing at least one trick five to nine times, and prior use of nicotine-free liquids when performing tricks. Participants will be not excluded based on race, sex, or cigarette smoking status. To accomplish the feasibility assessment, RTI will limit the number of recruitments to a target sample of 30 participants. The study team believes 30 participants is an appropriate number of participants to test data collection procedures and obtain a sufficient amount of data for analysis. Individuals will be excluded if they are younger than 18 or older than 29 years of age; pregnant; or suffer from claustrophobia, heart disease, an acute or chronic lung disease, or other chronic systemic illness. The RTI research staff are aware that the proposed methods for recruitment could potentially bias the sample; however, since the proposed study is a feasibility assessment, we are primarily concerned with recruiting the necessary number of participants and not with equal distribution of variables across participants.

### Participant Recruitment

E-cigarette users will be recruited via email and flyers posted at local vape shops, hookah bars, and other venues that young adult e-cigarette users may visit. The RTI research staff will visit the prospective venues and receive permission from the venue to hand out the recruitment flyers and ask venue staff to provide input on how best to reach the target population. Employees at AVAIL Vapor shops in Raleigh and Durham, North Carolina, will also conduct in-person recruitment by distributing flyers and study information sheets when interacting with customers at their stores. The recruitment materials will instruct potential participants to go to a website, which will direct them to the online screening survey to assess eligibility.

Recruitment will also occur online through social media ads and postings on websites such as Reddit, Facebook, and Instagram. For the social media recruitment, ads will not specify the study population or study goals in order to conform with ad platform requirements. Clicking on these social media ads will link participants to the screener survey where they will be presented with information about the study before being asked any screening questions.

Screened individuals deemed as eligible will be contacted by study staff via email, phone, or both to confirm eligibility and to schedule an appointment to visit the RTI lab. Prior to their appointment, participants will be informed that they will need to bring their own e-cigarettes and nicotine-free, hash oil-free liquids. Eligible individuals who are scheduled for a lab visit will also be contacted 24-48 hours before their visit via email to confirm the time and date. At the lab visit, staff will offer participants additional flyers if they are willing to share with others who might be interested in taking the screening survey and participating in the study.

### Informed Consent

Upon arrival at the RTI research facility, the RTI research staff will conduct the informed consent procedures. Written informed consent will be obtained from individuals who have completed the screening questionnaire and meet the screening criteria prior to enrollment in the study. As part of the consent process, participants will be informed that their participation is entirely voluntary and that they can stop at any time. If they stop before the end of the appointment, they will be compensated for activities completed (see Study Design section for information about compensation). Any questions asked by the participant will be answered before completing the informed consent process. The consent form will be signed and dated by the study participant and the RTI staff member conducting the consent procedures.

Since the participants’ sessions will be filmed, they will be asked to sign a standard RTI video release form. In addition, participants will be required to sign an attestation form stating that they meet the inclusion and exclusion criteria for the study, and women will be required to sign on the attestation form stating that they are not pregnant. As part of this study, a Hexoskin shirt will be worn; the Hexoskin is a shirt with biometric sensors worn directly against the skin. As such, participants will be asked if they currently have any open wounds on their chest or back where the Hexoskin shirt will be worn. If open wounds are present, the participant will not be allowed to enroll in the study.

### Study Design

After enrollment, participants will be fitted with the Hexoskin device, and each participant will then be assigned to complete either an ad-lib session or a vape tricks session. Each session will take place in RTI’s controlled exposure chamber, will last 20 minutes apiece, and will be videotaped. During the ad-lib session, participants will be instructed to puff on their e-cigarette as they normally would for the duration of the session. Participants will be offered the use of a television or tablet during the 20-minute ad-lib session.

During the vape tricks session, participants will be asked to stand in the chamber and perform a series of two to five vape tricks using their own device while being videotaped. Participants will be asked to perform tricks that they had an established history of performing prior to their participation in the study. Participants will be asked to repeat each trick four times, up to 20 tricks total, with a required minimum resting period of 30 seconds between trick attempts (ie, approximately one trick per minute). In the case of false starts or errors as they perform tricks, participants will not be allowed to attempt more than 30 tricks during a single 20-minute session. Participants will be allowed to rest for longer than 30 seconds or stop their participation in the study at any time. These time limits are based on the averages from prior studies of user puffing behavior. All participants will be compensated US $100, even if they stop before the end of the appointment.

### Proposed Variables by Data Source

#### Screener Survey and In-Person Interview

The online screening survey will assess age; cigarette smoking; health status, specifically whether the participant has any of 15 health issues that would result in exclusion; and how frequently they vape different e-cigarette device types (ie, nonrefillable, refillable without special features, and refillable with special features like temperature control). The screener will also assess individuals’ history of performing vape tricks, including whether they have done tricks in the past 3 months; which tricks they have performed (eg, *jellyfish*, *ghost inhale*, or other); the number of times they have tried those tricks; and what type of e-liquid they have used when doing tricks (eg, flavored or unflavored and with or without nicotine).

Before participants begin their first vaping session in the exposure chamber, study staff will conduct brief interviews. The interviews will cover the following topics: how participants would describe vape tricks to someone unaware of the behavior, participants’ first experiences with vape tricks, what tricks they know how to do, what tricks they will perform during the session, and whether they have ever competed in a vape tricks or *cloud chasing* competition (ie, trying to make the biggest cloud). Finally, the interviewer will ask the participant to describe the e-cigarette device they brought with them, including where they got the device, whether they built it, what features it has, and what kind of e-liquid they usually use with it.

#### Video Data Collection

Participants will be videotaped during the in-person interview, ad-lib vaping session, and vape tricks session. Qualitative coding will be conducted on ad-lib vaping and vape tricks videos to determine puff duration for both ad-lib and vape trick sessions. For the ad-lib vaping session, an analyst will record the time of the beginning of each puff. For the vape tricks session, the analyst will record the beginning of each trick puff, the time the inhale ends, and the time the trick ends. The beginning of the puff will be measured from the moment the e-cigarette is put up to the participant’s mouth. If the participant positions the e-cigarette in such a way that it is difficult to see the beginning of the puff, the analyst will also consider inhalation noise in their determination. The end of the inhale will be measured visually by looking at when a participant stops breathing in and by listening for the time at which noise from vaping ends.

#### Hexoskin

The Hexoskin device will record the heart rate, respiratory rate, tidal volume, minute volume, and any participant movement (eg, steps, acceleration, and position). Data collected by the Hexoskin device and recorded video data will be used to determine the inhalations recorded by the Hexoskin device that result from puffs taken on the user’s e-cigarette. These metrics will be summarized when comparing the data generated during the ad-lib session and the vape trick session across participants.

#### Micro-Personal Exposure Monitor

The Micro-Personal Exposure Monitor (MicroPEM; RTI International) is a small wearable sensor that collects up to 500 μg of particulate matter (PM) on a 25 mm polytetrafluoroethylene filter for gravimetric and speciation analyses. The device itself weighs less than 240 g and has a proprietary noise dampener in the flow control system, which effectively reduces the noise level to less than 3 dB above background at 1 m. The laser-based light scattering nephelometer collects real-time PM concentration data at up to 3-second resolution over a range of 3-15,000 µg/m^3^. The three-axis accelerometer monitors the frequency and intensity of movement for protocol compliance determination. The nephelometer and accelerometer data can be combined to calculate the potential inhaled dose. Quality control metrics continuously monitored are temperature, relative humidity, pressure drop across the filter, battery voltage, and sample flow rate.

#### Exposure Chamber

The RTI controlled exposure chamber, where all vape tricks and ad-lib sessions will be conducted, allows for control of temperature, humidity, and ventilation; the chamber size is 4.6 m x 3.4 m. Temperature and humidity in the chamber will be maintained at 23°C and 40% relative humidity, respectively. Ventilation will be controlled through an adjustable high-efficiency particulate air (HEPA)-filtered air plenum and conforms to American Society of Heating, Refrigerating, and Air-Conditioning Engineers (ASHRAE) specifications. The controlled ventilation rate will be set at a sufficient value to ensure that the air inside the chamber has been exchanged between subjects (ie, approximately 25 exchanges per hour). Electric power within the chamber will be supplied by nine 120 volts alternating current (VAC) 60 Hz outlets housed on dedicated circuits. We will place a model 3321 aerodynamic particle sizer (APS) spectrometer (TSI Inc) in the chamber with the participants. Participants will not interact with the APS and the APS will not alter the ambient air in the chamber. The APS is a high-performance, general-purpose particle spectrometer that measures both size and airborne concentration of the particles. The airflow through the APS passes through a high-efficiency filter to remove any particles before being exhausted into the room. We will place the APS on a table located 1 m from the participant using their e-cigarette device. The APS power cord will be connected to an electrical outlet behind the table and in the opposite direction from the participant. The APS pump will create an audible, noticeable hum that does not pose a risk.

### Study Outcomes

The primary outcome is to conduct a feasibility assessment comparing the heart rate, breathing rate, and activity metrics associated with those performing vape tricks to those associated with more typical ad-lib use of e-cigarettes. This will be accomplished through concurrent data collection using video- and sensor-based technology. In addition, a secondary outcome is the use of study results to program a smoking machine that will draw emission samples from e-cigarettes during a subsequent study phase that does not involve human subjects.

### Data Management

Data collected via electronic survey for screening purposes will include each participant’s email address and/or phone number, as well as their contact name to schedule the lab visit and deliver reminders. Access to the screening survey itself will be housed in RTI’s Enhanced Security Network, which is where Voxco is housed, constructed to the Federal Information Processing Standard (FIPS) to ensure security. At the conclusion of data collection, participant email addresses will be removed from study documents and destroyed. Contact information will be removed from all data files and destroyed to deidentify the dataset from the Enhanced Security Network files 12 months after data collection.

Each participant will be assigned a numeric study ID that will be used to designate all monitoring data collected through the Hexoskin device, the MicroPEM sensor, the exposure chamber, as well as the video and audio data. The data collected will be referenced only by these numbers in any laboratory documents, electronic database, and documented or published material. All collected data will be securely stored on RTI servers. Deidentified Hexoskin device data will also be stored on the online Hexoskin dashboard. Separately, any images from videos of participants for publication or reporting will be deidentified by cropping, blurring, or obscuring faces or other identifying information with black redaction boxes. Raw video and images will be destroyed at the conclusion of data analysis; only deidentified images or sample video will be stored for use in publications and reports.

### Data Analysis

#### Video Data Collection

An analyst will review the video from each participant to determine when their exhale ends by recording the time at which the vapor stream stops coming out of the participant’s mouth.

#### Hexoskin

The VivoSense (Vivonoetics) data analysis and software program will be used for complex respiratory analysis of the Hexoskin device data to generate quantitative profiles of both the ad-lib and vape trick sessions suitable for exposure modeling. Qualitative data from coded video will be used to isolate sensor data associated with ad-lib and vape trick sessions. Output from both modes of data collection will be matched and compared against one another to assess concordance.

#### Micro-Personal Exposure Monitor

MicroPEM PM2.5 (ie, diameter of less than 2.5 μm) and relative humidity data will be collected at a rate of one measurement every 10 seconds. Temperature will be logged every 30 seconds. This data will be plotted in a graph to show variability in PM2.5 as a function of temperature and relative humidity.

#### Exposure Chamber

Data from the APS will be analyzed to describe any changes in the aerosol concentration and particle size distribution following vape trick use of e-cigarettes.

## Results

Data analysis is pending and scheduled to take place in the fall of 2019.

## Discussion

### Overview

Although the national prevalence of performing vape tricks is not known at this time, some studies suggest that this may be a popular activity among e-cigarette users, especially among youth [19-21]. This study will be the first to examine puff topography and activity metrics associated with vape tricks and the first to characterize exposure to potentially harmful emissions resulting from that behavior. This study benefits from a mixed-methods approach that combines biometric sensor data and aerosol emissions data to best characterize how vape tricks differ from ad-lib vaping.

### Limitations

This study has several limitations. Because the protocol requires an in-person study visit, data collection will be geographically limited to the area around Research Triangle Park, North Carolina, potentially limiting generalizability. In addition, the recruitment sample runs the risk of potential bias since participants must be 18-29 years of age and have a history of performing vape tricks in the past 3 months; their breathing patterns, heart rates, and other metrics may not be equivalent to those who are younger or older or who are less experienced with vape tricks. For both the vape tricks and ad-lib vaping sessions, participants’ performance in the lab setting may not be identical to their performance in real-world settings. Further, use of the Hexoskin biometric shirt to conduct a topography study or to quantify respiratory exposure has not been previously validated.

### Conclusions

This research protocol describes the steps that will be utilized to characterize vape trick behaviors and subsequent aerosol emissions as part of a mixed-methods laboratory study. The results of this study should provide some initial data to help determine whether performing vape tricks poses a greater risk to health than ad-lib vaping. Ultimately, if a sufficient body of evidence suggests that vape tricks are more harmful than ad-lib vaping, tobacco control researchers and regulators might consider developing interventions to prevent this behavior. Future research using the methodology described here could be used to provide more accurate assessments of vaping behaviors obtained in a naturalistic setting, which could better account for sporadic but impactful variations in vaping behavior.

## Abbreviations

APS: aerodynamic particle sizer
ASHRAE: American Society of Heating, Refrigerating, and Air-Conditioning Engineers
FIPS: Federal Information Processing Standard
HEPA: high-efficiency particulate air
MicroPEM: Micro-Personal Exposure Monitor
PM: particulate matter
VAC: volts alternating current
